# Evaluating the Use of Tumor Bank DNA to Validate Genetic Factors Impacting Opioid Response in Patients with Advanced Cancer

**DOI:** 10.3390/curroncol33060363

**Published:** 2026-06-17

**Authors:** Christine L. Watt, Rebecca Lelievre, Gaelle Chopin Stukart Parsons, Caroline Vergette, Venus Chirip, Nadia Polskaia, Julie Lapenskie, Bryan Lo, Pearl Campbell, Asma Bankapur, Gareth Palidwor, James Downar

**Affiliations:** 1 Ottawa Hospital Research Institute, Ottawa, ON K1R 6M1, Canadagchopins@uottawa.ca (G.C.S.P.); cvergette@ohri.ca (C.V.); jdownar@toh.ca (J.D.); 2 Bruyère Health Research Institute, Ottawa, ON K1R 6M1, Canada; 3 Division of Palliative Care, Department of Medicine, University of Ottawa, Ottawa, ON K1R 6M1, Canada; 4 The Ottawa Hospital, Ottawa, ON K1H 8L6, Canada; 5 Department of Pathology and Laboratory Medicine, University of Ottawa, Ottawa, ON K1H 8M5, Canada

**Keywords:** palliative care, genetics, pain management, personalized medicine, opioids, retrospective cohort

## Abstract

Opioids are first-line therapy for cancer pain, yet up to 30% of patients fail to achieve adequate control at standard doses. Opioid response is partly genetically mediated, and understanding these factors may improve symptom management. This project aimed to assess the feasibility of using tumor bank DNA for pharmacogenetic analyses and to validate previously identified genetic variants associated with opioid response using existing genetic and clinical data. In this retrospective cohort study, clinical data (morphine equivalent daily dose, demographics) and genetic data (single-nucleotide polymorphisms) were analyzed across 31 candidate loci. Adult deceased patients with melanoma, colorectal, or lung cancer treated with opioids between 2016 and 2021 and with available tumor bank DNA were included. Patients without sufficient DNA or not deceased were excluded. Of 3503 potential samples, 502 met the inclusion criteria. The median morphine equivalent daily dose was 40 mg (range 1–2140 mg). Eleven loci across six genes may be associated with higher (*OPRM1*, *TAOK3*, *NFKBIA*, *COMT*, and *RHBDF2*) and lower (*COMT* and *GCH1*) opioid dose requirements (*p* < 0.05, not significant after Bonferroni correction). Ultimately, tumor bank DNA is a feasible resource for pharmacogenetic research. Identified loci may contribute to variability in opioid response and support future personalized pain management strategies.

## Simple Summary

Opioid medications such as morphine are commonly used to treat cancer-related pain, but many patients do not get enough relief even at usual doses. One reason may be differences in a person’s genes, which can affect how their body responds to these medications. In this study, we used stored tumor samples and medical records from patients with cancer to explore whether certain genetic differences are linked to how much opioid medication patients need for pain control. We found several genetic variations that were associated with higher or lower opioid doses. This study also shows that using existing tumor bank samples for this type of research is practical. These findings may help guide future studies and could eventually support more personalized approaches to pain management, where treatments are tailored to an individual’s genetic profile to improve comfort and quality of life.

## 1. Introduction

Pain is among the most common and distressing symptoms in patients with advanced cancer, with prevalence estimates of up to 70% over the course of illness [1]. Uncontrolled pain is strongly associated with insomnia, fatigue, depression, and nausea, and contributes to impaired function, decreased quality of life, and patient and family distress [2,3,4,5,6]. Opioid analgesics remain the cornerstone of therapy for moderate to severe cancer pain, and multiple international guidelines recommend their use as first-line treatment [1,7]. However, studies suggest that between 14 and 35% of patients experience undertreated pain [2,8], and the median time to achieve stable analgesia after opioid initiation may exceed three weeks, with some patients never reaching adequate relief before death [9]. Such delays are especially consequential in the palliative care setting, where quality of life is paramount and time to achieve symptom control may be limited.

It is difficult to predict which patients will struggle to achieve appropriate pain control [1,7,9]. Even after accounting for known clinical covariates such as age, sex, cancer type, and pain etiology, some patients require substantially higher opioid doses than expected, while others experience significant side effects at lower doses [9,10]. This variability in opioid response is, in part, genetically mediated [10,11]. In fact, twin studies estimate that genetic variance may account for as much as 60% of individual differences in opioid responsiveness [11].

Pharmacogenomic studies have identified single nucleotide polymorphisms (SNPs) that influence opioid pharmacokinetics and pharmacodynamics, including variants in *CYP2D6*, *OPRM1*, *ABCB1*, and *COMT* [12,13,14,15,16,17,18,19,20,21]. This research has advanced our understanding of how genetic variations influence individual responses to weak opioids such as codeine. However, the impact of genetic variations on the response to stronger opioids such as morphine and hydromorphone remains poorly defined [12,13,14,17]. Many reported associations have not been replicated across diverse populations, and much of the available evidence is derived from postoperative, pediatric, or experimental settings rather than advanced cancer or palliative care [22,23,24]. Furthermore, most existing studies rely on prospective recruitment and phenotyping, an approach that is resource-intensive and often not feasible in patients near the end of life [17].

There is a need for research that expands and validates our understanding of genetic contributors to opioid responsiveness in advanced cancer pain, using feasible study designs and diverse patient cohorts. Establishing such evidence would provide the foundation for a personalized approach to pain management—allowing clinicians to anticipate poor or delayed responses, tailor treatment strategies earlier, and ultimately improve quality of life for patients with advanced cancer.

This study seeks to assess the feasibility of using readily available genetic and clinical data to validate genetic factors associated with increased opioid use in cancer pain and examine genetic associations between 31 SNPs previously shown to be related to opioid response among patients with advanced cancer who experience pain.

## 2. Materials and Methods

Feasibility Outcomes: Feasibility of using tumor bank DNA was assessed by examining participant recruitment rates; final sample size; and the efficiency, quality, and variant calls generated through DNA sequencing of included samples.

Participants: Deceased, adult patients with lung cancer, colorectal cancer, or melanoma who used any amount of strong opioid (morphine, hydromorphone, oxycodone, methadone and fentanyl) and had readily available tumor biopsy DNA obtained between 2016 and 2021 were included in the study. When multiple samples were available for the same patient, only the first sample was included. Eligible participants were identified through the Eastern Ontario Regional Laboratory Association (EORLA) Anatomical Pathology Information System (APIS) database, a private tumor bank. Patients were excluded if they had insufficient DNA for sequencing or if they did not use any strong opioids during their illness. We excluded all patients who were not confirmed deceased as per medical records to ensure that DNA would not be required for decision making on further oncologic therapies.

Clinical Data: Demographic information, including age, sex and cancer type, was extracted from medical charts. Pain scores in clinician progress notes were recorded, if available. Peak morphine equivalent daily dose (MEDD) at any stage of illness was calculated as per current clinical practice [25,26], and the use of non-opioid adjuvant medications (ketamine, dexamethasone, pregabalin, etc.) was noted, when available.

Sequencing: Thirty-one candidate SNPs associated with opioid response in patients with cancer were identified through a literature review. The complete list of candidate SNPs can be found in Supplemental Table S1. Candidate SNPs were provided to Illumina Inc. (San Diego, CA, USA), a biotechnology company, which generated a custom amplicon (AmpliSeq) [27] panel. Genetic sequencing was completed using Next Generation Sequencing by StemCore Laboratories Genomics Core Facility (The Ottawa Hospital Research Institute, University of Ottawa, RRID:SCR_012601). Panels covering the regions containing each SNP of interest were designed using the DesignStudio Sequencing Assay Designer (Illumina Inc., https://www.illumina.com/products/by-type/informatics-products/designstudio.html, accessed 9 February 2023). Vendor-recommended settings for targeted AmpliSeq Panels for formalin-fixed, paraffin-embedded DNA were used in the panel design. Prior to library construction, DNA quality and quantity were assessed using the Fragment Analyzer (Agilent, Santa Clara, CA, USA) and the Qubit 3.0 (Thermo Fisher Scientific, Waltham, MA, USA), respectively. Only samples with the majority of DNA fragments measuring 200 bp or greater were used. Library construction was performed using the AmpliSeq Library Plus for Illumina Custom Panels (Illumina Inc.), using half-volume reactions with the manufacturer’s recommended protocol for formalin-fixed, paraffin-embedded DNA. For sequencing, the prepared libraries were normalized and pooled, then run on a NextSeq 2000 (Illumina Inc.) to achieve an average coverage of 50× for each SNP region.

Association Analysis: Variant association analysis using an additive effect model was conducted using PLINK (v2.00a5.14LM) to evaluate the relationship between genetic variants and opioid response. We considered MEDD as a continuous trait and post-hoc selected binary cutoffs of >120 mg, >200 mg and >300 mg, reflecting the top 20%, 10% and 5% of MEDD of the cohort, respectively. Analysis was also conducted against the log10-transformed MEDD score, as very few patients had very high MEDD values, and the log10 transformation allowed for a more normal distribution.

Where appropriate, multivariable linear or logistic regression models were applied to each phenotype to assess variant associations while accounting for covariates of age, sex and cancer type. Finally, a separate analysis was performed using methadone and ketamine as binary phenotypes, with the same covariates. Analyses were performed both with and without correction for multiple analyses (Bonferroni), owing to the hypothesis-generating objective of the study and the fact that our sample size was finite and unknown at the time of study design.

This retrospective cohort study received ethics approval via the Ottawa Health Sciences Network Research Ethics Board (approval code: OHSN-REB Protocol #20220298-01H; approval date: 17 June 2022). Given the nature of the study and the inclusion of only deceased participants, the requirement for informed consent was waived by the ethics board.

## 3. Results

### 3.1. Study Feasibility

Participant inclusion: A total of 3503 participant DNA samples from patients with lung cancer, colon cancer, or melanoma were identified in the EORLA APIS database. Of those, 2793 were excluded on initial chart review for failing to meet study inclusion criteria (patients with no opioid use, not deceased, multiple samples for the same patient). Another 208 participants were excluded, as samples did not have sufficient DNA banked for study use. Ultimately, 502 participant samples were provided for DNA sequencing and genotyping.

**Figure 1:**
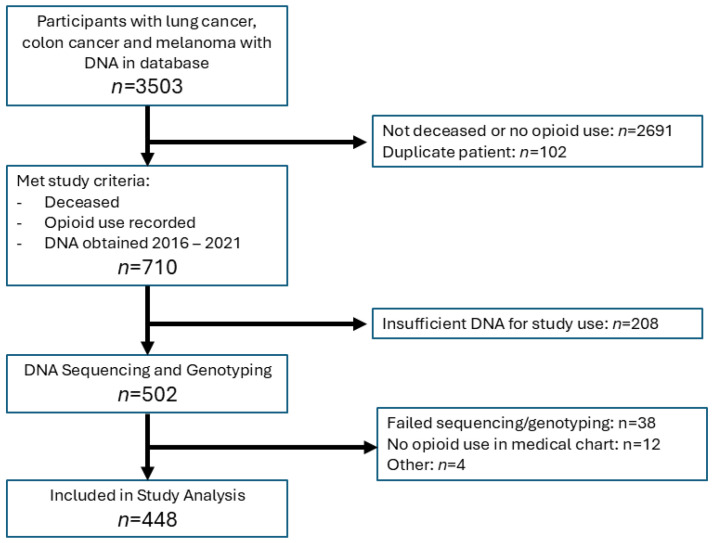
Record screening and participant inclusion flow diagram.

Sequencing: Of the 502 participant samples, 92% (*n* = 464) completed sequencing and SNP genotyping with appropriate quality and coverage (38 samples failed sequencing). Sixteen additional participants were excluded from the study during detailed chart review: 12 did not have any documented opioid use, and 4 samples were excluded for other reasons (non-cancer pain, opioid prescriptions for opioid use disorder, patient not formally listed as deceased in the medical chart). Ultimately, 448 participants were included in the study cohort. The process of record identification and participant inclusion is outlined in Figure 1.

Population Demographics: Table 1 lists the demographic and clinical characteristics of the study population. The population consisted of equal proportions of male and female patients, the majority (*n* = 320, 71%) with lung cancer. Nearly all patients were documented to have metastatic disease (*n* = 417, 93%). The median MEDD was 40 mg/day (range 1–2140 mg/day). Twenty-three patients (5%) had a MEDD of >300 mg, with five (1%) having a MEDD > 1000 mg. There was a significant correlation seen between age and log10 MEDD (r^2^ = 0.04, *p* < 0.001), but no significant associations between sex or cancer type and MEDD.

### 3.2. Candidate SNP Analysis

There was no deviation in the Hardy–Weinberg equilibrium for all SNPs. Candidate SNP analysis controlling for age, sex and cancer type identified seven SNPs across five genes that are associated with elevated MEDD at different thresholds (>120 mg (top 20% cohort), >200 mg (top 10% cohort), >300 mg (top 5% cohort) and MEDD as a continuous trait) (Table 2) and four SNPs in two genes associated with lower MEDD (Table 3) at >120 mg threshold only. No candidate SNP maintained significance after correcting for multiple tests. There was no statistical association between any of the candidate SNPs and the use of methadone as a primary opioid or adjuvant or adjuvant ketamine; however, sample sizes were low for both medications (*n* = 22 and 21, respectively). Complete variant association analysis results for all candidate SNPs are shown in Supplemental Table S2.

## 4. Discussion

Personalized medicine offers an opportunity to improve symptom management while minimizing adverse effects. In palliative care, achieving adequate pain control is often challenging, and side effects are common. A personalized approach could help identify patients who are more resistant to opioids, more susceptible to toxicity, or more likely to benefit from non-opioid strategies. Given the limited time many patients with advanced cancer have to achieve symptom relief, such insights could meaningfully improve quality of life for patients.

### 4.1. Study Feasibility

This study demonstrated the feasibility of using existing genetic data from tumor banks, raising the possibility of efficient study replication in other centers without prospective biospecimen collection. In palliative care and in patients with advanced illness, prospective study recruitment is often challenging and impacted by clinician gatekeeping, rapid deterioration, cognitive impairment and high study attrition [28,29]. Thus, it is often impractical or impossible to recruit highly symptomatic patients with advanced illness to any research study, let alone those requiring biospecimen collection. Using previously collected tumor bank DNA may be a novel approach to overcoming these recruitment challenges for future pharmacogenetic studies. To our knowledge, this is the first study to examine the feasibility of using existing tumor bank DNA to conduct retrospective genetic analysis for reasons outside of oncological care. We were able to achieve high rates of genotyping and variant calls (92% of samples with appropriate clinical and genetic DNA) for a fraction of the cost of a prospective study (less than $250 CAD per sample). Future studies should consider the use of readily available DNA from tumor biobanks if it can be linked to robust, complete clinical data.

### 4.2. Candidate Gene Analysis

This study identifies genetic variants of interest for ongoing study. *OPRM1* remains one of the most extensively studied genes in opioid pharmacogenetics. It encodes the μ-opioid receptor, which mediates analgesia and many opioid-related adverse effects, including nausea, respiratory depression, and cognitive impacts [13,30,31]. The rs1799971 variant is well characterized; however, *OPRM1* is highly polymorphic, with over 100 known variants contributing to interindividual variability [13,31]. *COMT*, another frequently studied gene, encodes catechol-O-methyltransferase, which regulates dopamine, epinephrine, and norepinephrine metabolism [20,30]. Although it does not directly affect opioid metabolism, *COMT* may influence pain perception and pain pathway activity. Prior studies have linked *COMT* variants to both increased and decreased opioid requirements, a pattern also observed in our cohort [28].

Additional candidate genes identified in this analysis are involved in signaling and inflammatory pathways relevant to pain processing, with more indirect effects. *GCH1* encodes an enzyme critical for tetrahydrobiopterin synthesis, which is involved in nitric oxide production and pain modulation [32]. *TAOK3* encodes a protein kinase that regulates multiple signaling cascades, including those associated with μ-opioid receptor pathways, and has previously been associated with increased morphine requirements in post-operative pain [33,34]. Inflammatory modulation is further represented by *NFKBIA*, which encodes an inhibitor of NF-κB, a transcription factor implicated in central sensitization and inflammation via the cyclooxygenase-2 pathway [35,36]. Finally, *RHBDF2*, identified through genome-wide association studies, regulates tumor necrosis factor–α and interleukin-6; however, its role in pain is not well understood [37]. Collectively, these findings highlight the complex, multifactorial genetic architecture of pain and opioid response beyond genes directly involved in opioid metabolism.

While two identified SNPs (rs1799971, *OPRM1* and rs4680, *COMT*) have been consistently reported across multiple studies [16,18,19,20,21,38,39,40,41,42], the remaining nine have been inconsistently replicated [24,32,33,37,43,44,45]. Additionally, 20 candidate SNPs did not reach any significance in our cohort. Discrepancies between study cohorts likely reflect population-level genetic differences in racially diverse cohorts, limited sample sizes and methodological variability, including patient population, opioids used and measures of pain and opioid response. Moreover, any study involving multiple comparisons is prone to spurious findings.

### 4.3. Strengths and Limitations

This study used readily available tumor DNA, which allowed for efficient study execution without the need for prospective recruitment or biospecimen collection. Although we were able to analyze data from a large proportion of eligible individuals, our sample size was limited by the quality and completeness of clinical documentation, an issue common in retrospective designs [46]. We also had to exclude participants if death was not explicitly documented in the medical record or if use of strong opioids could not be confirmed. In many cases, inadequate documentation of patient status or medication use resulted in exclusion. For example, although it could be reasonably inferred that a patient with advanced cancer who had no documented healthcare encounters for several years was deceased, the absence of explicit confirmation in the medical record necessitated exclusion. Additional samples were excluded due to DNA availability, as some were stored for many years and subject to repeated genotyping for novel oncologic therapies. Importantly, of the patients with adequate clinical data, 70% (*n* = 502/710) had sufficient DNA available for sequencing, and of these samples, nearly all completed sequencing and genotyping (92%, *n* = 448/502). This sequencing success rate is similar to other studies of formalin-fixed, paraffin-embedded samples [47]. Despite these limitations, this study represents one of the largest candidate gene analyses of opioid response in patients with advanced cancer.

This study used MEDD as the primary measure of opioid response, as the retrospective design limited the availability of pain scores, which were reported inconsistently, tumor burden, and data relating to toxicity and side effects. MEDD does not necessarily reflect pain control and can be impacted by patient factors (tolerance, organ dysfunction, goals of care and disease burden, etc.) as well as provider prescribing practices (type of opioid and doses) and goals of care. For example, high MEDD may not indicate poor pain control, but rather tolerance, while lower MEDD may indicate high susceptibility to side effects rather than satisfactory pain control. While MEDD is commonly used as a surrogate for opioid response [21,22,24,35,43,48], it remains an imperfect measure and should be considered thoughtfully.

In addition, while we were able to control for cancer type and sex, we were unable to reliably extract important clinical covariates such as metastatic location and tumor burden, type of pain (i.e., neuropathic, somatic), adjuvant medication use, and anti-cancer therapies, particularly at the time that peak MEDD was recorded in the patient chart. It was also not possible to extract patient ethnicity. This is of particular importance, as many SNP frequencies vary by ethnicity, including rs1799971 (*OPRM1*) and rs4680 (*COMT*) [49,50], and may impact generalizability of results to different regions. However, observed variant frequencies in our sample are consistent with those expected for our population when considering Statistics Canada and the genomAD database [51,52].

In this study, we used tumor DNA, assuming that this would be a reasonable surrogate for germline DNA for genotyping SNPs associated with opioid response. We acknowledge that cancer-associated somatic alterations, loss of heterozygosity, copy number alteration and tumor heterogeneity could affect genotyping accuracy. Several factors reduce the likelihood that these will substantially affect our analyses; variants are restricted to prespecified SNPs, and these variants are not associated with known recurrent oncogenic driver loci. Prior studies comparing matched tumor and germline-derived DNA have reported high concordance between tumor-derived and germline genotypes [53,54]. Somatic mutations affecting these loci are unlikely and, if present, would affect only a small number of samples and lead to random genotype misclassification or missing data. Unless such misclassifications were systematically associated with opioid response, they would be expected to bias associations towards the null rather than false positives [55]. This is a limitation of the study, and confirmation in cohorts with matched germline DNA would strengthen future analyses.

Importantly, none of the associations identified in our cohort remained statistically significant after correction for type I error, highlighting the challenges inherent in hypothesis-generating genetic analyses. Although the cost of DNA extraction and sequencing has decreased, it remains substantial, necessitating the testing of multiple SNPs to justify resource utilization. However, as the number of tested variants increases, so does the required sample size to detect meaningful associations after adjustment for multiple comparisons. For future hypothesis-driven studies, focusing on a smaller subset of the most biologically and clinically promising SNPs may enable more definitive interpretation and validation of genetic effects.

## 5. Conclusions

The use of tumor bank DNA is feasible and may represent an additional approach for future translational research. However, challenges with retrospective clinical documentation may limit sample size and available clinical data, and should be carefully considered. Using this novel approach, this study explored 11 SNPs in 6 candidate genes that may be associated with opioid use in advanced cancer; however, future research is warranted. Future studies on the genetic factors impacting opioid response will continue to drive more personalized palliative care and improve patient symptom control.

## Figures and Tables

**Table 1:** Study cohort demographics.

Patient Characteristic	*n* (%)
Sex	
Male	229 (51%)
Female	219 (49%)
Cancer Type	
Lung	320 (71%)
Colorectal	98 (22%)
Melanoma	30 (7%)
Morphine Equivalent Daily Dose	
0–30 mg	197 (44%)
31–60 mg	91 (20%)
61–90 mg	39 (8%)
91–120 mg	34 (7%)
121–150 mg	20 (4%)
151–180 mg	12 (3%)
180–210 mg	13 (3%)
211–240 mg	6 (1%)
241–270 mg	8 (2%)
271–300 mg	5 (1%)
>300 mg	23 (5%)
Ketamine Use	21 (5%)
Methadone Use (primary opioid or adjuvant)	22 (5%)

**Table 2:** SNPs of significance associated with higher MEDD use across MEDD thresholds of >120 mg (top 20% cohort), >200 mg (top 10% cohort), >300 mg (top 5% cohort) and MEDD as a continuous trait.

SNP ID	GENE	MEDD > 120		MEDD > 200		MEDD > 300		MEDD Continuous Trait	
		ß-Value	*p*-Value	ß-Value	*p*-Value	ß-Value	*p*-Value	ß-Value	*p*-Value
rs1799971	*OPRM1*	0.350	0.113	0.407	0.159	**0.743**	**0.039**	**40.290**	**0.024**
rs795484	*TAOK3*	**0.328**	**0.048**	0.204	0.355	0.019	0.949	24.592	0.063
rs2233419	*NFKBIA*	0.167	0.470	**0.592**	**0.035**	**0.802**	**0.020**	27.817	0.145
rs2233417		0.132	0.520	**0.506**	**0.049**	0.570	0.091	18.248	0.268
rs3138054		0.191	0.361	**0.547**	**0.038**	0.619	0.0671	20.8642	0.218
rs12948783	*RHBDF2*	0.253	0.225	**0.572**	**0.028**	−0.167	0.687	−0.480	0.977
rs165728	*COMT*	0.103	0.748	0.233	0.567	0.321	0.537	**67.739**	**0.009**

**Bold**: *p* < 0.05.

**Table 3:** SNPs of significance associated with lower MEDD use across MEDD thresholds of >120 mg (top 20% cohort), >200 mg (top 10% cohort), >300 mg (top 5% cohort) and MEDD as a continuous trait.

SNP ID	GENE	MEDD > 120		MEDD > 200		MEDD > 300		MEDD Continuous Trait	
		ß-Value	*p*-Value	ß-Value	*p*-Value	ß-Value	*p*-Value	ß-Value	*p*-Value
rs10483639	*GCH1*	**−0.468**	**0.031**	−0.308	0.2778	−0.162	0.655	−19.025	0.220
rs3783641		**−0.492**	**0.031**	−0.272	0.354	−0.569	0.190	−29.630	0.067
rs8007267		**−0.521**	**0.022**	−0.349	0.241	−0.837	0.084	−28.768	0.072
rs4680	*COMT*	**−0.334**	**0.034**	−0.107	0.617	−0.423	0.146	−11.167	0.382

**Bold**: *p* < 0.05.

## Data Availability

Anonymized data supporting the findings of this study are available from the corresponding author upon request.

## Supplementary Materials

The following Supporting Information can be downloaded at: https://www.mdpi.com/article/10.3390/curroncol33060363/s1, Table S1: Candidate single nucleotide polymorphisms (SNPs); Table S2: Variant Association Analyses for Candidate Single Nucleotide Polymorphisms (SNPs).

## Abbreviations

The following abbreviations are used in this manuscript:

DNA	Deoxyribonucleic acid
SNP	Single Nucleotide Polymorphism
MEDD	Morphine Equivalent Daily Dose
EORLA	Eastern Ontario Regional Laboratory Association
APIS	Anatomical Pathology Information System
