# Supplementary material for: Evaluating the Use of Tumor Bank DNA to Validate Genetic Factors Impacting Opioid Response in Patients with Advanced Cancer
Source: Curr Oncol. 2026 Jun 17;33(6):363. doi: 10.3390/curroncol33060363 (PMC13298010; doi:10.3390/curroncol33060363)
Supplement: Supplementary file 1 [file curroncol-33-00363-s001.zip › curroncol-4308087-supplementary.pdf]

Supplemental Table S1. Candidate single nucleotide polymorphisms (SNPs). Candidate SNPs identified through literature review. **Bold:** Candidate SNPs with significant association to opioid daily dose in current study. *Italic: studies with negative results for SNP.*

Gene	SNP ID	Nucleotide change	References
OPRM1 - Opioid receptor Mu	**rs1799971**	A>G	Gong et al 2013, Cajanus et al 2014, Hajj et al 2017, Campa et al 2008, Klepstad et al 2004, Matic et al 2017, Wong et al 2024 [16,18–21,40,41] *& Klepstad et al 2011, Nishizawa et al 2022*[22,24]
	rs9322446	G>A	Yennurajalingam et al 2021[43]
	rs2270459	C>A	
	rs62052210	A>G	
	rs1319339	T>C	
	rs34427887	C>G/T	
	rs9479759	C>T	
	rs2003185	C>T	
	rs636433	A>C/G	
COMT-Catechol-o-methyltransferase	**rs4680**	G>A	Rakvag et al 2005, 2008, Matsuoka et al 2017, Matic et al 2017, Wong 2024 [20,21,38, 39, 42] *& Klepstad et al 2011, Nishizawa et al 2022*[22,24]
	rs4818	C>G	Ferreria et al, unpublished[44]
	rs2020917	C>T	Yennurajalingam et al 2021[43] *& Klepstad et al 2011*[22]
	rs9306234	A>C	Yennurajalingam et al 2021[43]
	**rs165728**	C>G/T	Yennurajalingam et al 2021[43]
	rs4646316	C>G/T	Yennurajalingam et al 2021[43] *& Nishizawa et al 2022*[24]
	rs35478083	T>A/C	Yennurajalingam et al 2021 [43]
ABCB1 - ATP binding cassette subfamily B	rs1045642	A>G	Gong et al 2013, Campa et al 2008, Qin et al 2024 [15,16,19] *& Klepstad et al 2011, Nishizawa et al 2022*[22,24]
IL6 - Interleukin 6	rs1800795	C>G	Reyes-Gibby et al 2008[45] *& Nishizawa et al 2022*[24]
GCH1 - GTP Cyclohydrolase	**rs8007267**	C>T	Lotsch et al 2010, Nishizawa et al 2022 [24,32]
	**rs3783641**	T>A	Lotsch et al 2010, Nishizawa et al 2022 [24,32]
	**rs10483639**	G>A/C/T	Lotsch et al 2010 [32] *& Klepstad et al 2011, Nishizawa et al 2022*[22,24]
TAOK3 - Tao kinase 3	rs1277441	G>A/C	Gutteridge et al 2018[33]
	**rs795484**	T>C	Gutteridge et al 2018[33]
ANGPT1 – angiopoetin 1	rs1283671	C>T	Nishizawa et al 2022[24]
	rs1283720	G>A	Nishizawa et al 2022 [24]
RHBDF2 – Rhomboid 5 homolog 2	**rs12948783**	G>A	Galvan et al 2011[37] *& Nishizawa et al 2022*[24]
NFKBIA - NF-kappa-B inhibitor alpha coding gene	**rs2233419**	G>A	Yennurajalingam et al 2021[42]
	**rs2233417**	C>T	Yennurajalingam et al 2021[42]
	**rs3138054**	G>G/T	Yennurajalingam et al 2021[42]
	rs1050851	G>A	Yennurajalingam et al 2021[42]
ARRB2- Beta arrestin 2	rs1045280	C>T	Yennurajalingam et al 2021[42] *& Klepstad et al 2011*[22]

Supplemental Table S2. Variant Association Analyses for Candidate Single Nucleotide Polymorphisms (SNPs). Complete analyses of all candidate SNPs across morphine equivalence daily dose (MEDD) thresholds of >120mg (top 20% cohort), >200mg (top 10% cohort), >300mg (top 5% cohort) and MEDD as a continuous trait. **Bold:** SNPs of significance.

SNP ID	Gene	n	MEDD >120		MEDD >200		MEDD >300		MEDD Continuous Trait	
			ß-value	P-value	ß-value	P-value	ß-value	P-value	ß-value	P-value
rs1319339	*OPRM1*	442	-0.050	0.832	0.275	0.350	0.243	0.542	-3.270	0.859
rs1799971	*OPRM1*	443	0.350	0.113	0.407	0.159	**0.743**	**0.039**	**40.291**	**0.024**
rs9322446	*OPRM1*	443	-0.020	0.938	-0.454	0.248	-0.444	0.408	-22.950	0.255
rs9479759	*OPRM1*	443	0.300	0.309	-0.026	0.952	-1.553	0.134	-11.269	0.638
rs2003185	*OPRM1*	439	-0.057	0.731	-0.069	0.759	-0.290	0.329	-8.085	0.529
rs636433	*OPRM1*	444	0.245	0.358	-0.204	0.611	**-1.836**	**0.076**	-21.045	0.331
rs34427887	*OPRM1*	446	-0.416	0.347	0.246	0.606	-0.052	0.942	-8.465	0.781
rs2270459	*OPRM1*	434	-0.092	0.748	-0.314	0.462	-0.590	0.341	-23.392	0.286
rs62052210	*OPRM1*	441	-0.040	0.891	-0.277	0.519	-0.516	0.407	-22.438	0.313
rs1800795	*IL6*	442	-0.085	0.617	0.055	0.810	0.156	0.605	4.160	0.749
rs1045642	*ABCB1*	447	-0.107	0.527	-0.159	0.488	-0.143	0.637	-6.703	0.609
rs1283671	*ANGPT1*	445	-0.125	0.632	0.135	0.678	0.262	0.524	8.176	0.676
rs1283720	*ANGPT1*	445	0.094	0.697	0.041	0.900	0.169	0.685	14.159	0.458
rs795484	*TAOK3*	444	**0.328**	**0.049**	0.204	0.356	0.020	0.949	**24.595**	**0.063**
rs1277441	*TAOK3*	441	0.266	0.110	0.152	0.493	-0.078	0.799	20.886	0.114
rs2233419	*NFKBIA*	437	0.168	0.470	**0.592**	**0.035**	**0.803**	**0.021**	27.818	0.145
rs2233417	*NFKBIA*	441	0.132	0.520	**0.506**	**0.049**	**0.570**	**0.091**	18.248	0.268
rs3138054	*NFKBIA*	440	0.191	0.361	**0.547**	**0.038**	**0.619**	**0.067**	20.864	0.218
rs1050851	*NFKBIA*	438	0.144	0.471	**0.483**	**0.058**	0.534	0.109	20.557	0.201
rs10483639	*GHC1*	444	**-0.468**	**0.031**	-0.308	0.277	-0.162	0.655	-19.026	0.220
rs3783641	*GHC1*	439	**-0.492**	**0.031**	-0.272	0.354	-0.569	0.190	**-29.630**	**0.067**
rs8007267	*GHC1*	436	**-0.521**	**0.022**	-0.349	0.241	**-0.837**	**0.084**	**-28.768**	**0.072**
rs1045280	*ARRB2*	414	0.088	0.603	-0.131	0.573	0.028	0.923	-10.902	0.419
rs12948783	*RHBDF2*	441	0.253	0.225	**0.572**	**0.028**	-0.167	0.687	-0.480	0.977
rs2020917	*COMT*	442	**-0.362**	**0.072**	-0.368	0.183	**-0.712**	**0.075**	-20.660	0.162
rs4818	*COMT*	438	-0.284	0.108	0.046	0.844	-0.236	0.456	-8.869	0.508
rs4680	*COMT*	436	**-0.334**	**0.040**	-0.107	0.617	-0.423	0.146	-11.167	0.382
rs4646316	*COMT*	429	-0.241	0.223	-0.152	0.573	-0.171	0.632	-10.833	0.469
rs9306234	*COMT*	387	-0.220	0.198	-0.003	0.990	-0.283	0.372	-11.078	0.407
rs35478083	*COMT*	443	0.583	0.104	0.483	0.284	0.344	0.551	24.199	0.442
rs165728	*COMT*	444	0.103	0.748	0.234	0.568	0.322	0.537	**67.740**	**0.009**
